# Patient-physician communication in advanced kidney disease: a narrative review

**DOI:** 10.1007/s40620-024-02176-3

**Published:** 2025-01-14

**Authors:** Mohadese Golsorkhi, Niloufar Ebrahimi, Mehrbod Vakhshoori, Sayna Norouzi, Amir Abdipour

**Affiliations:** 1 Psychiatry Department, Brookdale Medical Center, Brooklyn, NY USA; 2 Department of Medicine, Division of Nephrology, Loma Linda University Medical Center, Loma Linda, CA USA

**Keywords:** Patient-physician communication, Nephrology, Dialysis, Kidney failure, Palliative care

## Abstract

The increasing prevalence of kidney failure highlights the crucial need for effective patient-physician communication to improve health-related quality of life and ensure adherence to treatment plans. This narrative review evaluates communication practices in the context of advanced kidney disease, focusing on the frameworks of shared decision-making, advanced care planning, and communication skills training among nephrologists. The findings highlight the significant gaps in patient-physician communication, particularly in the domains of advanced care planning, shared decision-making, and dialysis withdrawal. When communication in such situations falls short, it affects patient satisfaction and outcomes. Effective communication is essential for managing advanced kidney disease, requiring a patient-centered approach that includes patients, families, and healthcare providers in care planning. This review advocates for a more integrated, empathic approach to healthcare interactions, emphasizing the importance of including patients, their families, and healthcare providers in a collaborative model based on goals of care. Moreover, it highlights the role of patient-reported outcome measures and patient experience of care in assessing the quality of care in dialysis settings. Here, we call for enhanced training programs regarding communication skills for physicians and emphasize the need for systemic changes to foster patient-centered communication.

**Graphical abstract:**

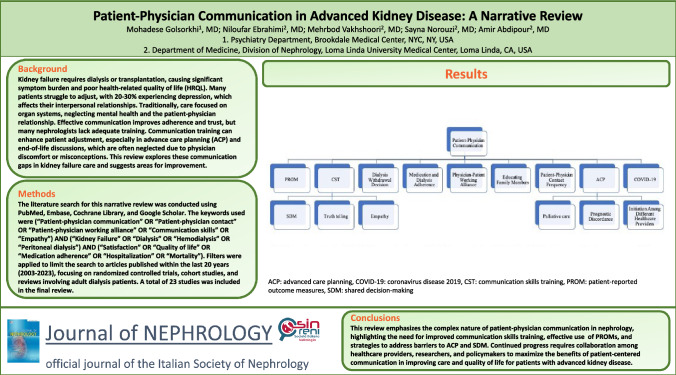

## Introduction

The number of individuals diagnosed with kidney failure in the United States peaked at 808,000 in 2020, marking a surge of 107% since 2000 [1]. Kidney failure necessitates kidney replacement therapy (KRT) such as dialysis or transplantation and is associated with substantial symptom burden and poor health-related quality of life (HRQL) [2]. Patients find it challenging to cope with their kidney failure, adjust to dialysis sessions three times a week, or transition to a life with a transplanted kidney, and many see this as a traumatic event [3, 4]. It is not surprising that 20–30% of kidney failure patients experience depression, [5] and their interpersonal interactions are affected [6].

Conventionally, medical practice was directed towards organ systems rather than considering the impact of the disease on the patient’s mental health, highlighting the significant role of the patient-physician relationship in clinical practice [7]. Effective communication between physicians and patients has been shown to improve treatment adherence and build trust, facilitating constructive relationships [8]. Good communication skills are crucial to patient care; however, many nephrologists do not receive adequate training regarding this matter. Data show that patients with chronic kidney disease (CKD) stages 3–5 have varying levels of understanding about their disease and treatment options and the risks/benefits associated with each option. [9] Additionally, individuals with advanced kidney failure report inadequate end-of-life discussions with their doctor [10].

Communication skill training improves physicians’ ability to communicate effectively, assist patients in adjusting to their illness, and even prepare them for end-of-life care [11, 12]. Advanced care planning implies continuous communication among the patient, family members, and healthcare providers regarding the appropriate future medical care in case the patient becomes incapable of making their own decisions [13]. It considers the patient’s goals, values, and preferences regarding end-of-life care to achieve control over their future healthcare decisions. Common barriers in discussing advanced care planning include physicians neglecting to start uncomfortable conversations with patients and inaccurately presuming or disregarding patients’ preferences [14]. This could come from the physician’s lack of confidence in shared decision-making and misconceptions about the appropriateness of starting these discussions [15]. In this review, we explored how kidney failure patients perceive communication with their doctor, the importance of shared decision-making in clinical practice, advanced care planning in dialysis patients, and dialysis withdrawal decisions with a specific focus on the elderly population. We also identified gaps in this area to guide future improvements in care for this demographic.

### Literature search method

The search strategy for this narrative review was conducted using the following databases: PubMed, Embase, Cochrane Library, and Google scholar. The keywords used were (“Patient-physician communication” OR “Patient-physician contact” OR “Patient-physician working alliance” OR “Communication skills” OR “Empathy”) AND (“Kidney Failure” OR “Dialysis” OR “Hemodialysis” OR “Peritoneal dialysis”) AND (“Satisfaction” OR “Quality of life” OR “Medication adherence” OR “Hospitalization” OR “Mortality”). Filters were applied to limit the search to articles published within the last 20 years (2003–2023), focusing on randomized controlled trials, cohort studies, and reviews involving adult dialysis patients. A total of 23 studies were included in the final review.

### Understanding patient perspectives

**Fig. 1:**
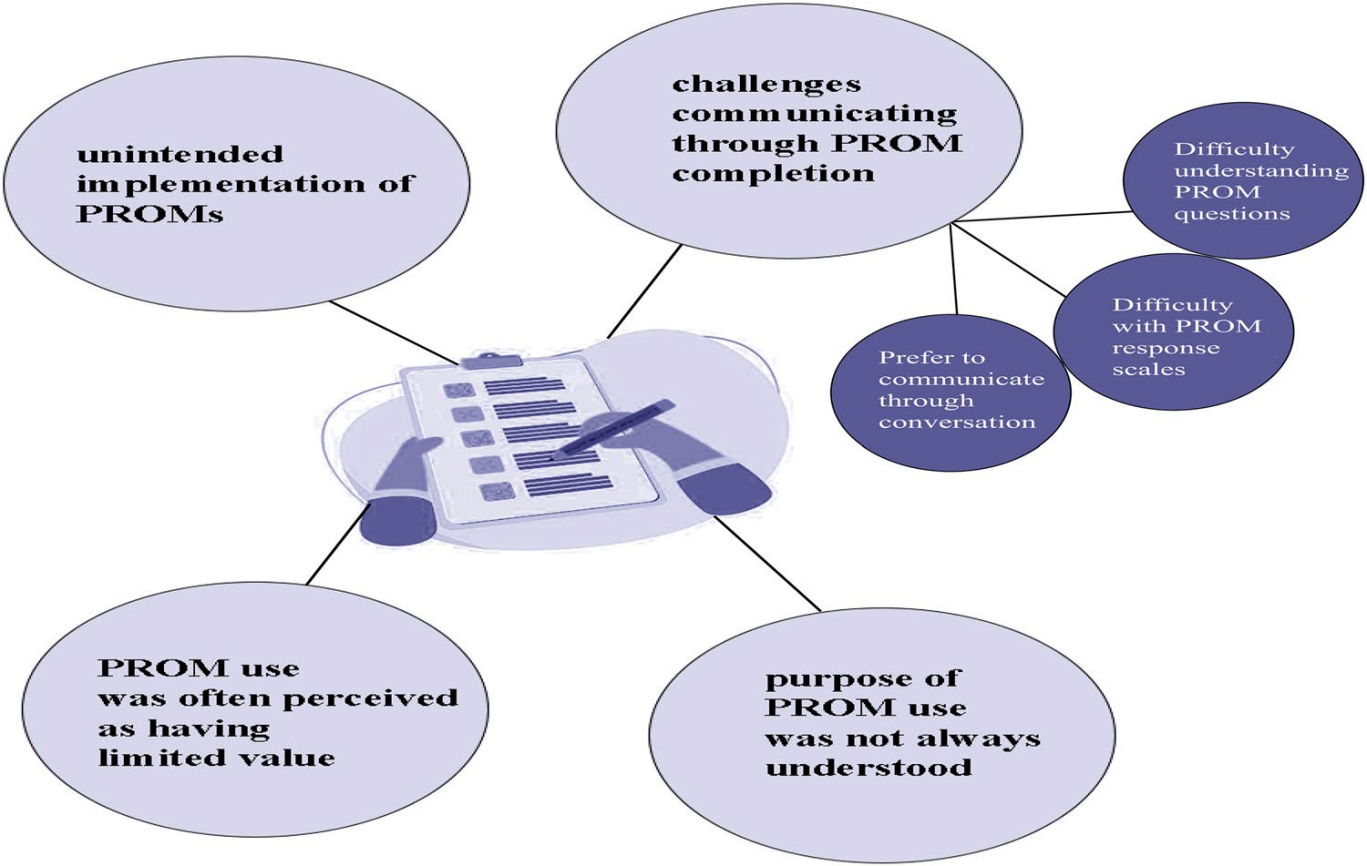
PROM use did not improve patient-clinician communication

Patient-reported outcome measures are standardized tools designed to help patients appraise and report healthcare outcomes such as physical, mental, functional status, and social well-being [16]. The patient-reported outcome measure is used in many areas, including dialysis settings, and it is reported to enhance communication between patients and clinicians in order to improve the process of care and facilitate shared decision-making. [17, 18] Schick-Makaroff et al. [19] designed a longitudinal mixed method research to evaluate how routine use of patient-reported outcome measures in hemodialysis (HD) settings in northern Alberta could influence patient-clinician communication. The authors used data from the Evaluation of Routinely Measured Patient-Reported Outcomes in Hemodialysis Care (known as EMPATHY) trial. Eligibility criteria were patients undergoing long-term HD treatment and willingness to participate in the study. They used a modified communication assessment tool [20] to analyze how routine use of a patient-reported outcome measure affects patient-clinician communication. A total of 417 kidney failure patients completed the communication assessment survey (mean age: 64 years, males: 57%, not working: 87%). The study also included interviews with ten patients and eight nurses to hear their opinions about the routine use (every two months) of patient-reported outcome measures in HD settings. The results showed that the routine use of patient-reported outcome measures did not improve patient-clinician communication within 12 months (*P-value* = *0.21*). Despite the intended purpose of patient-reported outcome measures to standardize patient-reported information for more individualized follow-up care, there was a misunderstanding regarding its application among patients. Many patients were unaware of the purpose of filling the survey out and wondered if it was “just part of a regular protocol” or for “research purposes.” Patients also reported having difficulty “quantifying their experiences with symptoms,” and some preferred to start the conversation with their clinician in case they had concerns. Last but not least, patients reported that a patient-reported outcome measure has limited value as it sometimes measures items that are not related to patients and their specific needs. In conclusion, while patient-reported outcome measures are intended to standardize patient-reported outcomes and improve individualized care, their routine use in dialysis settings, as shown in this study, highlights challenges such as patient misunderstanding, difficulty in symptom quantification, and limited relevance to individual needs, which undermine their effectiveness in enhancing patient-clinician communication. Figure 1 shows the challenges present regarding the implementation of patient-reported outcome measures.

### Communication skills training in nephrology

#### Truth-telling and shared decision making

Truth-telling and shared decision-making are essential communication strategies in the healthcare system that can be strengthened through targeted training programs. These communication skills workshops play a vital role in enhancing the quality of care for patients with advanced kidney disease.

**Fig. 2:**
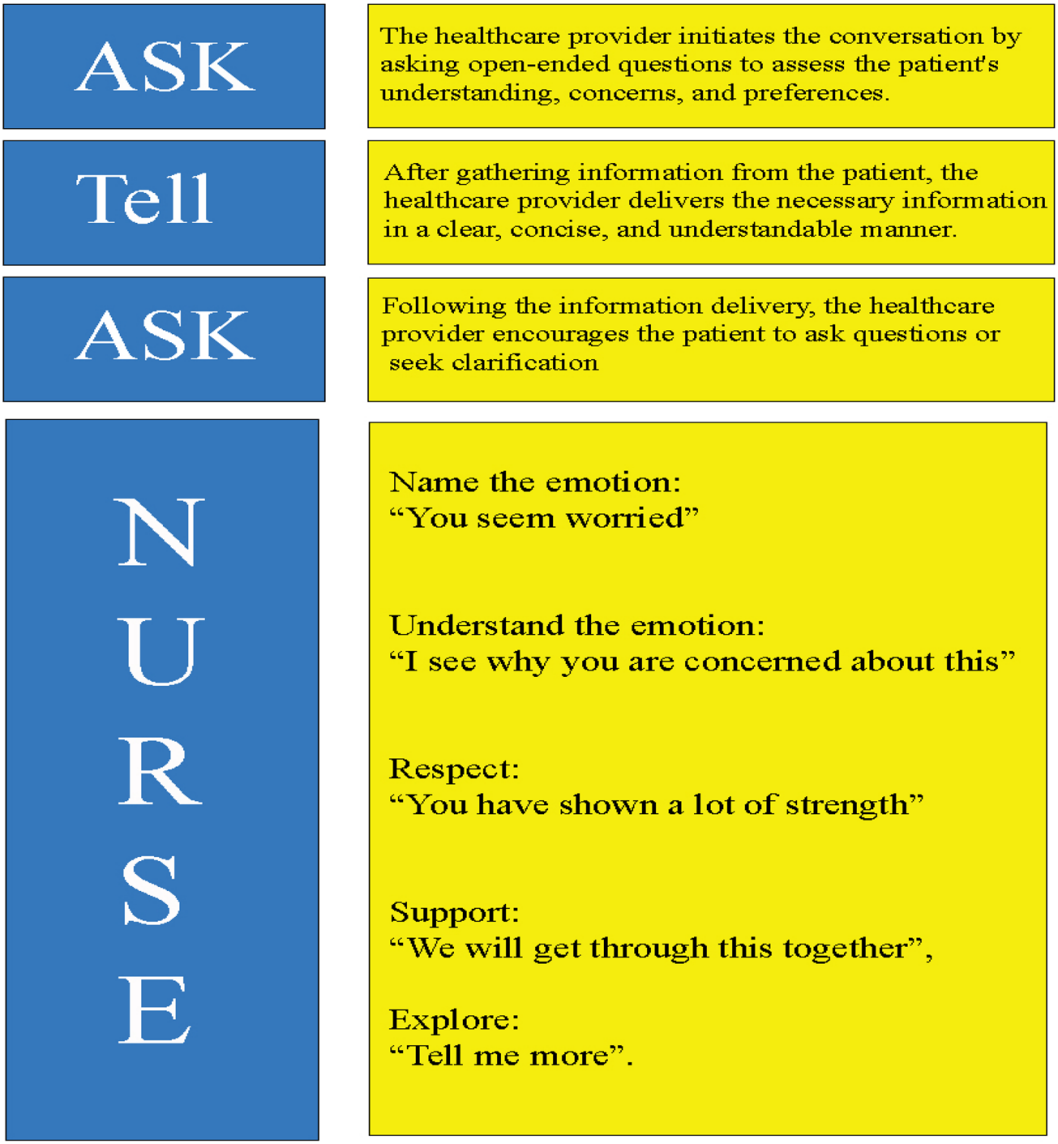
NephroTalk workshop models

Jane Schell et al. developed a communication skill workshop called “NephroTalk” to help nephrology fellows prepare for difficult conversations with their patients about end-of-life kidney care [21]. The curriculum was modeled after OncoTalk, a successful communication skills program with previously documented efficacy in the fields of oncology, palliative care, and geriatrics [22]. The workshop consisted of two parts: delivering bad news and defining goals of care with patients progressing to advanced stages. These two topics were reported to be the most challenging and anxiety-provoking subjects for physicians when communicating with patients [23]. Two common issues concerning communication skills in this context are “truth-telling” and “shared-decision making.” [24]. In delivering bad news, nephrology fellows were introduced to giving information using the Ask-Tell-Ask model (Ask: assess understanding, Tell: after learning what the patient knows, the physician can better tell the information in a way that addresses patient concerns and needs, Ask: check understanding) and addressing emotional responses using the NURSE acronym (Fig. 2) In defining goals of care, fellows were taught how to use open-ended “big picture” questions to understand better the patients’ goals and to offer a treatment option based on their preferences. The workshop evaluated its efficacy by pre- and post-training surveys measuring learner satisfaction and perceived preparedness. Nineteen nephrology fellows finished the survey, with 15% identifying as White. Among the 19 nephrology fellows, 50% were male, and 41% were in their initial year of the fellowship program. All responders indicated that communication skills are “important” or “very important” to becoming a “great nephrologist.” The analysis showed that mean perceived preparedness significantly increased for all communication challenges, including delivering bad news, expressing empathy, and discussing dialysis initiation or withdrawal (*P-value* < *0.010*). Some fellows commented that they learned to “listen more intently, limit the use of medical terminology, and give patients more time to express feelings” with this workshop.

By considering the importance of discussing treatment options with patients, shared decision-making emerged as an approach to share the best available options with patients and respect their autonomy to achieve informed preferences [25]. Fang et al. [26] designed a communication skills training program for educating nephrologists and senior nephrology nurses about truth-telling and shared decision-making in clinical practice. Participants were randomly assigned to the intervention group (n = 45) and control group (n = 46). The intervention group completed an online communication skills training course in addition to the usual in-service training, while the control group engaged in the standard in-service training that focused solely on fundamental communication skills. Communication skills training comprised two modules: truth-telling and shared decision-making. Each module provided a theoretical background along with video examples of interactions with kidney failure patients. Participants completed two surveys at three different times: before the intervention (T0), two weeks after the intervention (T1), and four weeks after the intervention (T2). Surveys included “Confidence in Communication with Patients” (21 self-reported questions used to test truth-telling) and the “Combined Outcome Measure for Risk Communication and Treatment Decision-Making Effectiveness” (COMRADE, 20 self-reported questions). They also asked about participant satisfaction with and intention to recommend kidney failure communication skills training to their colleagues. The mean age of participants was 41.6 years, and most were female (80%). The most significant proportion comprised registered nurses (64.8%) with bachelor’s degrees (47.3%). The findings indicated that the intervention group’s confidence levels were significantly greater than those of the control group at T1 (*P-value* = *0.006*). However, no significant differences were observed at any point in time with the COMRADE assessment. A high satisfaction rate was noted, with 95.6% of participants expressing satisfaction with the kidney failure communication skills training and more than 90% indicating they would recommend the course to peers. Although this study was limited and only found a short-term moderate effect in improving truth-telling confidence, online course training has been on the rise since the coronavirus disease 2019 (COVID-19) pandemic and future studies must carefully design interactive modules and evaluate its efficacy.

#### Empathy

Empathy is defined as one’s ability to listen to, share, understand, and respond to the experiences of others [27]. Showing empathy by physicians can enhance health outcomes, including life quality and pain management [28]. A study showed that the empathy displayed by physicians could lead to better HbA1C and low-density lipoprotein levels among individuals with diabetes mellitus (*P-value* < *0.010*). [29] Forgiveness, which is defined as the act of releasing recurrent anger, hostility, and the desire for revenge, has been associated with both the happiness and well-being of individuals [30]. In a hypothesis model by Ye et al., the correlation between empathy and HRQL was tested in 457 HD patients, and the moderating effect of forgiveness was evaluated. Empathy level was assessed using the Interpersonal Reactivity Index-C, forgiveness level was measured by the Heartland Forgiveness Scale, and HRQL was evaluated using the Kidney Disease Questionnaire. The participants’ mean age was 57.63 years; 57.99% were male, and 52.30% had primary glomerular disease. The correlation analysis indicated a significant association between all study variables. Empathy had a significantly direct effect on forgiveness (*P-value* = *0.012*) and HRQL (*P-value* = *0.017*). Forgiveness also exerted a notable direct effect on HRQL (*P-value* = *0.008*). As a result, the study confirmed the direct effect of empathy on HRQL and established forgiveness as a moderating factor in the empathy-HRQL relationship. These findings put more insights into the effect of empathy on patients’ lives by emphasizing the important role of forgiveness in this relationship [30].

Alice Doreille et al. [31] reported findings from a cross-sectional study that evaluated the influence of training programs on physician–patient communication in nephrology clinical practice. A four-hour educational workshop was offered to 46 nephrology trainees by experienced nephrologists and mental health professionals. The session started with a testimony video from a patient with kidney failure who had initially refused to acknowledge his kidney failure, which resulted in starting dialysis in the context of a metabolic crisis. The patient discussed the reason behind his denial and dialysis rejection, illustrating a common scenario where patients are insufficiently prepared for dialysis. The session continued with three consecutive case studies: 1. Informing a patient, known for their consistent follow-up and trust in medical advice about their kidney failure and the need for KRT; 2. A patient with a diagnosis of autosomal dominant polycystic kidney disease (ADPKD) exhibiting denial and refusing treatment while experiencing significant anxiety, and 3. An urgent case where a patient was admitted to the hospital due to previously undiagnosed kidney failure who required emergency dialysis. The trainees were invited to evaluate their satisfaction with the training session, its potential effect on their clinical practice, and their levels of empathy (using the Jefferson Scale of Physician Empathy or JSPE) at three points: before the session, immediately after, and several months later. Among the participants, 97% deemed the training to be essential and very useful, and all except one trainee believed the course would influence their approach to clinical practice. Furthermore, 76% of the trainees reported that the training had a noticeable impact on their medical practices in the following weeks. The training session resulted in a significant enhancement in empathy scores among participants immediately following the course (*P-value* = *0.005*) and was sustained several months thereafter (*P-value* = *0.040).* The training session helped nephrologists to reconsider how to deliver a kidney failure diagnosis. The findings showed that a single training session in the form of role-playing can have immediate and long-lasting effects on the clinical practices of nephrologists and improve one’s ability to put themselves in their patients’ shoes.

### Advanced care planning

Advanced Care Planning is an essential, continuous conversation involving the patient, family members, and healthcare professionals. This collaborative process is dedicated to discussing and planning for the patient’s future medical care in scenarios when they may no longer be capable of making their own healthcare decisions [15]. On the other hand, advance directives are formal, legal documents that specifically outline a patient’s healthcare preferences, such as living wills or durable power of attorney, to guide decision-making when the patient can no longer communicate their wishes. Understanding the distinction between these two concepts is crucial for ensuring that both preferences are respected, and legal requirements are met in their care [15]. An end-of-life decision is a challenging situation, and patients who decide to withdraw from treatment before death will experience a significant burden of physical and psychological symptoms. In such circumstances, hospice care plays a vital role in actively managing symptoms and providing emotional support and bereavement services to their families. Currently, hospice care for patients with kidney disease is significantly underused, such that only 20% of patients with kidney failure use hospice care services, compared to 55% of patients with cancer and 39.1% of patients with congestive heart failure [32].

### Palliative care in nephrology

Nephrologists play a pivotal role in assisting patients with making informed decisions about continuing dialysis or switching to conservative treatment. Integrating palliative care with the standard of care is reported to help patients manage their symptoms effectively, adapt to their illness, and prepare for end-of-life care [33]. There has been an increased demand for palliative care in recent years, with a growing focus on enhancing the development of primary palliative care skills among healthcare providers.

Koncicki and Schell [34] have delineated these primary palliative care skills into four sections:

1. Using prognostic tools to choose patients who may benefit from conservative management.

Prognostic discussions can help patients understand their clinical picture and get ready for their future plans. Although patients value conversations about their prognosis, these discussions occur infrequently [10]. Research indicates that the lack of engagement in prognosis discussions causes kidney failure patients to be reluctant to make treatment decisions during periods of clinical decline. Consequently, these patients often receive more intensive end-of-life care or fail to take advantage of hospice care [35]. According to the Renal Physician Association (RPA) guidelines, patients with two or more of the following prognostic indicators are at higher risk of experiencing suboptimal outcomes on dialysis: age > 75 years, high comorbidity score (*Charlson Comorbidity Index, The Renal Epidemiology and Information Network, Thamer et al. mortality risk score, and integrated 6-month mortality tool*), poor functional status or disability, and severe chronic malnutrition (e.g., serum albumin <2.5 g/dL) [36]. Furthermore, the clinicians’ insight is a determining factor in prognosis evaluation. It is further supported by a study that validated the use of the “surprise question” in prognosis evaluation (would I be surprised if this patient died in the next year?) [37].

2. Disclosing prognostic information to patients who may not do well on dialysis.

The RPA has crafted a framework to guide shared decision-making around the initiation or cessation of dialysis therapy [36]. This approach regards the discussion as a collaboration between two experts: the healthcare provider, who is an expert in medical knowledge, and the patient, who holds expertise in their own goals and values. The key component of this framework is the “Ask-Tell-Ask” technique, which encourages physicians to first inquire about the patient’s information preference (“What kind of information would you like to know?”). Following the provision of information, it is important to circle back and confirm the patient’s understanding regarding the information given (“That was a lot of information. Can you tell me what you will take away from this discussion?”). It is important to consider using appropriate language and giving time to patients to process the information given.

3. Incorporating patients’ values and goals into the treatment plan.

After conveying the prognosis information, it is time to shift the conversation to elicit the patient’s goals and values into the treatment plan (“When you think about the future, what do you hope for?”). This step involves describing two potential options: opting for dialysis or choosing conservative management (“Is it more important to you to live as long as possible,

despite suffering or live without suffering for a shorter period of time?”). This can be achieved by understanding the patient’s hopes and expectations, addressing any concerns about the future, and exploring any limitations in the quality of life in which the patient would consider withdrawing from life-supporting therapies such as dialysis. This process ensures that the treatment plan not only addresses the medical aspects of the patient’s condition but also aligns with their personal values and quality of life preferences.

4. Preparing patients for transitions and end-of-life trajectory.

Dialysis-specific triggers that require reassessing the treatment goal involve the patient’s inability to tolerate dialysis, refractory symptoms despite undergoing dialysis, or complications associated with access or the treatment itself. Such discussions are more likely to be successful when the patient/family and the healthcare provider mutually recognize change. This conversation occurs best by naming that “things have changed.”

It is advised that the nephrology team initiate the conversation about palliative care treatment, and the extent to which these discussions are being carried out depends on the experience and comfort of the clinician. When physicians feel no longer comfortable continuing the discussion, managing refractory complications, or addressing cases of “futility,” consultation with a palliative care specialist is recommended [38].

### Prognostic discordance between patients and physicians

Despite preferences for pain relief and quality of life, many dialysis patients spend their last month in hospitals, with underutilization of hospice care. Moreover, 60% of dialysis patients expressed regret regarding initiating treatment, and a significant portion withdrew from KRT, emphasizing the need for realistic prognostic discussions [39]. Studies suggest that kidney failure patients, like cancer patients, tend to be more optimistic about survival than their nephrologists [40]. A total of 66 patients from a HD center in New York were recruited for a study to evaluate patient-physician prognostic awareness and discordance [41]. An objective prognostic estimate was assessed using the prognostic index evaluated by Thamer et al. Patients’ willingness to discuss life expectancy and end-of-life care with their nephrologists was also evaluated. Four nephrologists also completed a separate questionnaire on patient life expectancy and discussions regarding prognosis. Demographic data showed a mean age of 60.8 years and a mean dialysis duration of 3.55 years. Most patients were White (73%), with hypertension (35%) and diabetes (18%) being the major causes of kidney failure. The majority of patients (95%) believed HD would prolong their lives and demonstrated optimistic prognostic views, with 81% confident that they would live more than a year and 67% believing they would live for more than five years. Regarding the goal of care, 45% of patients prioritized life extension, while 55% preferred that pain and discomfort be relieved even if it meant a shorter duration of life. Out of the patients surveyed, 53% reported that their nephrologist had discussed their prognosis. Of them, 47% expressed that they did not want to engage in such a discussion with their nephrologist. After defining prognostic discordance as > 20% difference between the patients and their nephrologists, 23% of patients were categorized as being in prognostic concordance with their nephrologist, while 77% were identified as being in discordance. Concordant patients tended to be younger and had fewer comorbidities. This study showed that patients with kidney failure overestimate their prognosis. Overestimating prognosis while patients’ goal of end-of-life care is more oriented towards pain relief and comfort can lead patients to undergo unnecessary, aggressive interventions and delay hospice care. These findings highlight the need for proper prognostic discussions between nephrologists and their patients.

### Facilitating advanced care planning

Comprehensive patient care extends beyond the medical and technical aspects of dialysis, highlighting the crucial role of advanced care planning [42]. Facilitated advanced care planning refers to a thorough process of understanding, communicating, and deliberating end-of-life care preferences between patients, their families, and healthcare providers. Facilitated advanced care planning is not a routine part of dialysis units, with only 6–35% of patients diagnosed with kidney failure having advanced directives in place [43].

Sara Davidson [44] designed an ethnographic study using personal interviews with 24 kidney failure patients who were willing to discuss their experiences and thoughts on advanced care planning. These patients, ranging in age from 44 to 88 years, primarily suffered from kidney failure due to diabetes and hypertension (62%). The interview topics included patients’ interest in talking about advanced care planning, what information to receive, and the role of physicians, patients, and their families or caregivers in facilitated advanced care planning. The study reported that the willingness to engage in conversations about advanced care planning varied significantly among patients and their family members. Advanced care planning was considered to be an important part of their medical care when they had a clear idea of how it would benefit them. As patients experienced fear and uncertainty about their future, the role of talking about their prognosis and disease progression became more prominent, especially at the initiation of dialysis. Participants expressed the belief that physicians bear the responsibility for initiating discussion about prognosis and guiding the process: “They will let me know when it is time”, or “I would hope that healthcare providers are sufficiently trained to inform patients at the right time what to expect. Not wait until the very last minute.” They did not view the advanced care planning process as just an information-giving session and acknowledged the crucial role of empathic listening. Most of the participants expressed a preference for engaging in shared decision-making with their healthcare team and their families.

### Factors influencing patients’ initiation of advanced care planning

**Fig. 3:**
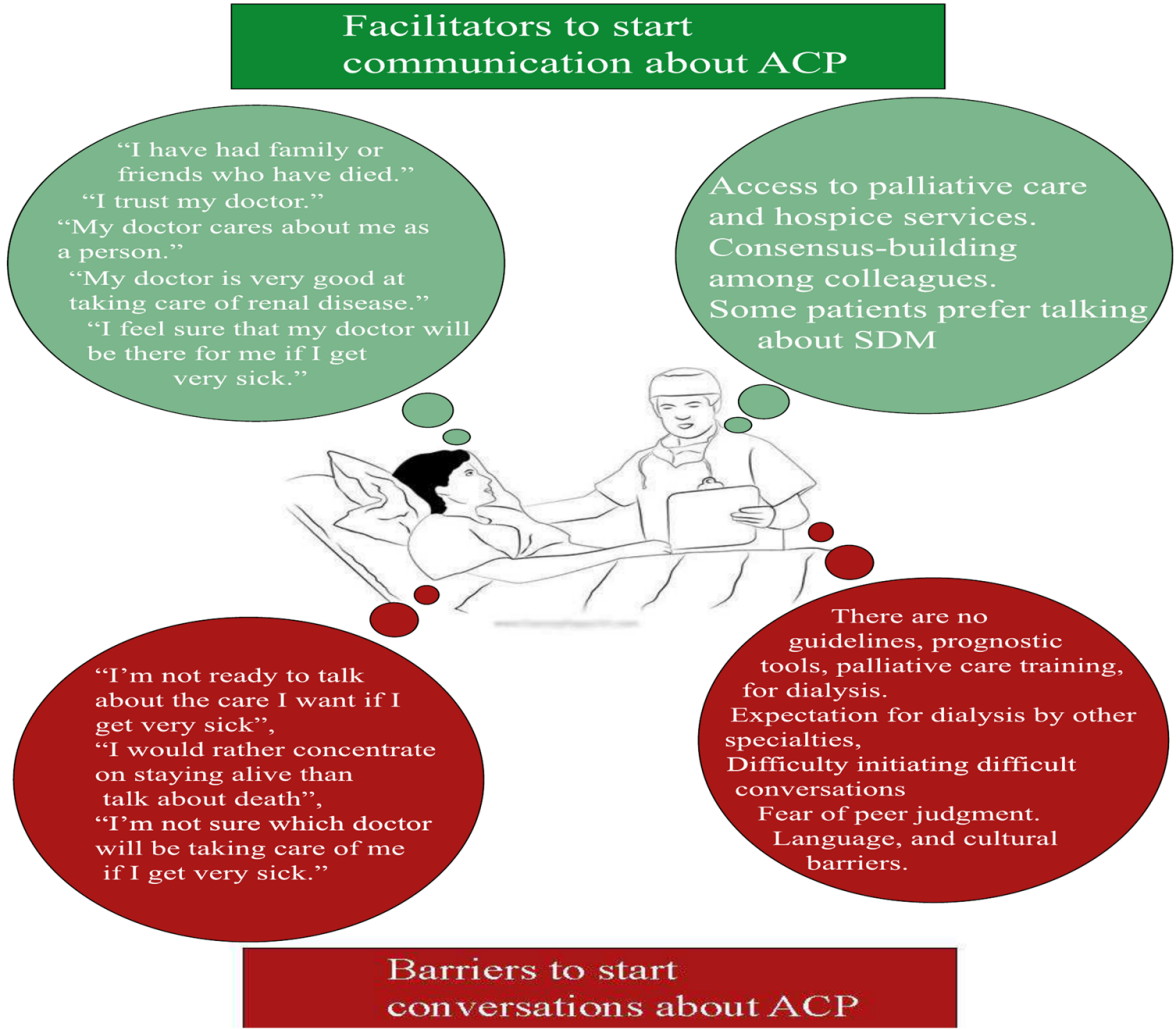
Facilitators and Barriers in starting ACP

In a cross-sectional study on 80 dialysis patients in the Netherlands, the quality of patient-physician communication regarding end-of-life care, barriers, and facilitators in such discussions was assessed [45]. The authors used the Quality of Communication (QOC) [46] and Barriers and Facilitators Questionnaires (BFQ) [47] (comprising 15 barriers and 11 facilitators for end-of-life care communication). The mean age of the patients was 62.2 years, and 60% were male. On the QOC questionnaire, patients rated the nephrologists’ overall general communication skills as high (median score 8), but the end-of-life communication skills were rated low (median score 1.1). Low means scores stemmed from patients reporting that items concerning end-of-life care were not communicated. These included elements such as the possibility of patients getting sicker (70%), how long patients have to live (85%), what dying might be like (91.3%), spiritual or religious beliefs (87.5%), or involving patients in treatment discussions about their care (63.8%). The barriers most commonly cited for communication about end-of-life care included “I’m not ready to talk about the care I want if I get very sick,” “I would rather concentrate on staying alive than talking about death,” and “I’m not sure which doctor will be taking care of me if I get very sick.” Conversely, the most frequently mentioned facilitators for communication about end-of-life care were “I have been very sick”, “I have had family or friends who have died,” “I trust my doctor,” “My doctor cares about me as a person,” “My doctor is very good at taking care of renal disease,” and “I feel sure that my doctor will be there for me if I get very sick.” (Fig. 3)

### Factors influencing physicians’ initiation of dialysis withdrawal

The widespread availability of dialysis therapy has transformed its use from a selective treatment to a routine procedure for chronic kidney failure. However, there is growing recognition that certain patients, such as those over 75 years old with conditions like dementia or heart disease, may not benefit from dialysis and may even experience a worsened quality of life [48]. Decision-making regarding proceeding or withdrawing dialysis varies among healthcare providers globally and over time. Vanessa et al. [49] tried to understand the underlying factors driving these differences, focusing on nephrologists’ perspectives in the United States and England, where conservative management programs are established. The study included 59 interviews: 18 with English nephrologists and 41 with American nephrologists. Most participants were White males under 45 years old. On average, they had 14.2 years of experience in post-nephrology training. English nephrologists practiced within a closed system, while American nephrologists were in academic and private practice settings with varying payment structures. Nephrologists’ perceptions were categorized into barriers and facilitators at the system level concerning preceding and withdrawing dialysis therapy.

System-level factors included the health care system, the culture of medicine, and societal culture. Based on the results, there are barriers and facilitators identified in foregoing dialysis as follows:

### Barriers to foregoing dialysis

There are no guidelines, prognostic tools, palliative care training, communication, or financial incentives for dialysis. “We really don’t know who’s going to do well and who doesn’t. So, I always err on the side of—at least give them a trial, see how it goes.” [American nephrologist].

“We have very little, if anything, about patient report[ed] outcome measures, quality of life measures. That’s a big deficiency across the system.” [English nephrologist].

Expectation for dialysis by other specialties, difficulty initiating difficult conversations, fear of peer judgment. “The patient was vented for more than six months, and she was described as a vegetative state. They were doing everything for her except that when she needed dialysis, they said, ‘Okay, you tell [the family] there’s no indication for dialysis.’” [American nephrologist].

Societal expectation for intervention and misperceptions about chronic kidney failure and dialysis. “If the family says, ‘We want everything done,’ and automatically that means that you have to do dialysis. For me, it’s that we want everything done, but for somebody who is declining. We wouldn’t do brain surgery if we knew it wasn’t going to save them.” [American nephrologist].

### Facilitators to foregoing dialysis

• Access to palliative care and hospice services. “If [48] option is taken by the family, then we provide the supportive care clinic that’s got two nurses, a dedicated two consultants who run a sort of referral and review system. So, if you do choose not to dialyze, you’ll be seen repeatedly by a dedicated team who you’ll get to know.” [English nephrologist].

• Consensus-building among colleagues. “I think overall, it felt like it was a well-managed process. It didn’t come as a surprise to anyone because we saw that his clinical status had changed. When dialysis was no longer a benefit but a burden to him, we, as a care team, met and had extensive discussions about it.” [American nephrologist].

**Fig. 4:**
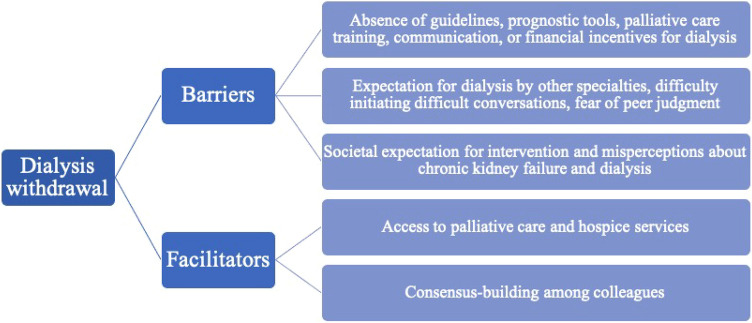
Factors associated with dialysis withdrawal decision

Figure 4 summarizes potential barriers and facilitators related to dialysis withdrawal decisions.

### Initiating advanced care planning among different healthcare providers

Initiating conversations about advanced care planning differs among healthcare professionals. Social workers and nephrologists often start such discussions with kidney failure patients but not with nephrology nurses [50]. Few nurses see themselves in the role of starting such dialogues with patients [27]. A research project involving renal physicians (N = 51), renal nurses (N = 461), and renal social workers (N = 13) in Singapore investigated the knowledge, viewpoints, and experience of advanced care planning among healthcare practitioners [15]. The authors developed a questionnaire that included 41 items divided into demographic information, knowledge, attitudes, and experiences related to advanced care planning. Most of the respondents in all groups had worked in the renal field for less than five years; 68.6% were practicing in the public sector and 17.7% in private clinics. Only 53.8% of the subjects engaged with the knowledge section, and the average score of nephrologists (8.0 out of 9.0) and social workers (8.3) was comparable to each other and higher than that of nurses (6.3). Of those who did not attempt the knowledge section, 91.7% were nurses. The questions with the highest true response were related to the need for good communication skills for advanced care planning discussions, appropriate timing of advanced care planning, and considering the patients’ life values and preferences. The question with the lowest score was related to advanced care planning as a legal document rather than a process.

The attitude section consisted of questions about the negative effect of advanced care planning discussion on patients and their families, barriers to discussing advanced care planning, benefits of advanced care planning, and views on patients’ autonomy. All groups agreed with the potential benefits of advanced care planning discussions. In contrast to physicians and social workers, two-thirds of nurses agreed with or were undecided about the statement that “prolongation of patient’s life is more important than honoring patient’s request to refuse life-sustaining treatment.” The nurses were also more concerned about the perceived barriers to discussing advanced care planning, and the social workers were less concerned. On a deeper level, participants who expressed concerns about upsetting their patients during discussions about advanced care planning were also more prone to worrying about the patient losing hope (r = 0.53, *P-value* < *0.001*) or feared being blamed by the family for the patient’s choices (r = 0.37, *P-value* < *0.001*). Not having the skills for discussing advanced care planning and a lack of understanding of advanced care planning were significantly associated with a lack of emotional strength to support patients in advanced care planning (r = 0.57, P-value < 0.001, and r = 0.49, P-value < 0.001, respectively).

The response to “Do you consider advanced care planning discussion as one of your roles?” varied among different groups. Most physicians and social workers replied positively, but only 37.1% of the nurses would see themselves in this role. Most nurses (90.3%) and physicians (66.6%) never discussed advanced care planning with their patients, compared to 53.9% of social workers. In this study, the top three barriers for physicians were the fear of upsetting the family, lack of time, and the assumption that the patients were not ready for such conversations yet.

### Medication and dialysis adherence

Current research on non-adherence in dialysis patients has mainly focused on prevalence and descriptive correlates, neglecting psychosocial factors and quality of life [51]. Non-adherence to HD is defined as skipping at least one dialysis session or shortening one or more sessions by more than 10 min per month. Non-adherence to both dialysis sessions and medication is reported to be associated with more hospitalization and mortality [52, 53]. Various psychosocial disturbances such as depressive symptoms, perception of social support, and patient-physician working alliance affect adherence to dietary and fluid guidelines among kidney failure patients [54].

### Physician–patient working alliance and treatment adherence

The patient-physician working alliance is a construct that fosters agreement, effective communication, and the development of trust and rapport between the patient and the healthcare provider [55]. Literature shows that patients dealing with chronic medical conditions who express a solid working alliance with their physicians are more adherent to treatment and show higher levels of satisfaction [55–57]. To establish an effective patient-physician working alliance, the attachment between doctors and patients is crucial. To evaluate the effect of the patient-physician working alliance on adherence in a dialysis setting, Fuertes and colleagues enrolled 107 kidney failure patients in their study [54]. Of them, 65.4% were male, their mean age was 55, and their average time on HD was five years. Participants were queried regarding their patient-physician working alliance, evaluating cognitive and emotional aspects of goal setting, shared decision-making, and trust. Correlation analysis revealed that the patient-physician working alliance has a positive correlation with adherence (r = 032, *P-value* < *0.001*), quality of life (r = 0.34, *P-value* < *0.001*), satisfaction (r = 0.74, *P-value* < *0.001*), and outcome expectations (r = 0.55, *P-value* < *0.002*). Path analysis also showed that the patient-physician working alliance indirectly predicts adherence, quality of life, patient satisfaction, and outcome expectations.

A recent study by Reyes et al. [58] hypothesized that social support and the patient-physician working alliance were expected to be positively associated with treatment adherence, satisfaction, and HRQL in kidney failure patients. A total of 95 eligible participants were enrolled, consisting of 53 males (56%) and 42 (44%) females. The average participant was about 58 years of age. Almost all participants were of lower socioeconomic status, with 81% of the sample reporting an income of less than or equal to $30,000 a year. Patients were asked to fill out the Beck Depression Inventory-II, the Medical Outcomes Study (MOS) questionnaire on social support (containing 19 items that evaluate the perceived availability of social support), the patient-physician working alliance inventory (12-item measure includes three subscales: (a) Emotional bond, (b) Agreement on treatment goals, (c) Agreement on treatment tasks), medical patient satisfaction questionnaire, general adherence measure, and World health organization quality of life-BREF (WHOQOL-BREF).

The working alliance showed a positive and significant correlation with self-reported treatment adherence (r = 0.32*, P-value* = *0.001*), treatment satisfaction (*r* = *0.57, P-value* < *0.001*), quality of life (r = 0.36, *P-value* < *0.001*), and social support (r = 0.23, *P-value* = *0.028*). The study revealed that patients with high levels of depression in the context of kidney failure and HD treatment tend to struggle with treatment adherence, lack of social support, and reported poor quality of life. Therefore, it is crucial to assess depression among these patients regularly. Routine assessment of depression and social support, along with a collaborative working alliance between patients and healthcare providers, are recommended to improve treatment adherence and outcomes for this population.

### Educating family members

Family plays a significant role in treatment success, as adherence often occurs in the home environment. Educating both patients and their families is essential for reducing complications and promoting treatment adherence. Zolfaghari et al. [59] compared the effects of family-centered and patient-centered education on reducing hypotension and muscle cramps in HD patients. Hypotension and muscle cramps were common complications during dialysis and significantly impacted patients’ quality of life and mortality rates. This study enrolled 30 individuals in each group. The mean age was similar between the patient-centered (47.41 years) and family-centered (48.16 years) groups. The most common cause of HD in both groups was diabetes, with most patients having undergone dialysis for 1–5 years. In the patient-centered group, educational content was presented to the patients themselves, and in the family-centered group, one member of the family attended the academic sessions in addition to the patient. During the second and fourth weeks following the intervention, educating patients and their families led to a significant reduction in the occurrences of hypotension. In the family-centered group, 73.3% and 63.3% of individuals did not experience this complication during the second and fourth weeks post-intervention, respectively. In contrast, in the patient-centered group, 73.3% and 60% of participants experienced sudden hypotension during the second and fourth weeks post-intervention, respectively. Family-centered education significantly reduced the frequency of both complications compared to patient-centered education. Consequently, physicians are advised to engage in shared conversations with patients and their families to improve treatment adherence and prevent complications.

### Frequency of patient-physician contact

The limited time and labor resources available in healthcare affect patients’ frequency of clinic visits. On the other hand, efficient patient education and empowerment can foster self-care and decrease the need for visiting doctors [60]. So far, there is limited evidence on whether the frequency of patient-physician contact can enhance the quality of care and outcomes among dialysis patients. A large cohort study enrolled 735 HD patients to evaluate the correlation of patient-physician contact with treatment adherence, hospitalization, quality of life, and mortality. The mean frequency of patient-physician contact was assessed and categorized into three groups (low: monthly or less frequent; intermediate: between monthly and weekly; and high: more than weekly). They reported that patients within the lowest patient-physician contact group had higher odds of non-adherence than the highest group (odds ratio: 2.89, 95% CI: 1.01–8.29). However, no significant difference was found in terms of hospitalization, quality of life, or mortality [61].

A second study showed that less frequent patient-physician contact among 678 dialysis patients treated at clinics with less frequent physician contact exhibited lower odds of meeting most targets, specifically for albumin (low: adjusted OR = 0.83, 95% CI = 0.55–1.25; intermediate: adjusted OR = 0.62, 95% CI = 0.42–0.93; reference: high) and dialysis dose (low: adjusted OR = 0.26, 95% CI = 0.08–0.89; intermediate: adjusted OR = 0.67, 95% CI = 0.20–2.27). However, they had higher odds of achieving the hemoglobin target (low: adjusted OR = 1.94, 95% CI = 1.24–3.04; intermediate: adjusted OR = 1.89, 95% CI = 1.27–2.83) [62]. Ying Xu et al. [63] assessed how patient-physician contact affects health outcomes in 307 peritoneal dialysis (PD) patients. The frequency of patient-physician contact was recorded as high frequency (monthly or more often), intermediate frequency (every 1–3 months), or low frequency (every 3–6 months). The primary outcomes were baseline clinical indices (hemoglobin, albumin, blood urea nitrogen, creatinine, calcium, phosphorus, and eGFR), dialysis adequacy and nutrition (daily protein and energy intake). A total of 127 patients (41.3%) were in the high-frequency group, 136 (44.3%) in the intermediate-frequency group, and 44 (14.3%) in the low-frequency group. Throughout the study, patients with varying frequencies of clinic visits had similar baseline demographics and clinical characteristics. The frequency of patient-physician contact did not affect the survival of PD patients (*P-value* = *0.37*), and there was no observed impact of visit frequency on mortality when adjusted for known risk factors. The authors also reported no differences in Charlson comorbidity score or baseline clinical indices such as blood urea nitrogen, creatinine, calcium, and eGFR between the three groups (*P-value* > *0.05*). However, patients in the low-frequency group had lower mean Hb and total Kt/V levels but higher serum P levels than patients in the intermediate- and high-frequency groups (*P-value* < *0.05*). The average measures of nutrition (serum albumin, daily protein, energy intake, and lean body mass) showed no variation across the three groups (*p-value* > *0.05*). However, anemia and hyperphosphatemia control must be strengthened in patients with low-frequency patient-physician contact.

In conclusion, while the frequency of patient-physician contact may influence certain clinical outcomes, such as treatment adherence and specific biochemical targets, it does not appear to significantly impact overall survival or major health outcomes such as hospitalization, quality of life, or mortality in dialysis patients, suggesting that targeted interventions beyond patient-physician contact frequency may be needed to optimize patient care.

### Challenges in communication during the COVID-19 pandemic

The COVID-19 pandemic has resulted in significant loss of life in the United States, with over 300,000 deaths reported. Patients with kidney disease face heightened risks from COVID-19, necessitating careful management by nephrologists. Nephrologists treating COVID-19 patients encounter new diagnostic dilemmas and psychological stressors. To address these challenges, interdisciplinary collaboration among nephrologists, palliative care specialists, intensivists, and ethicists is crucial [64].

Researchers have documented different cases highlighting communication challenges in nephrology during the COVID-19 pandemic.

#### Case #1 (challenges in communication)

A 60-year-old Spanish-speaking man with chronic kidney disease and morbid obesity was hospitalized with respiratory distress and tested positive for COVID-19. He deteriorated rapidly, requiring intensive care unit (ICU) admission, intubation, and continuous KRT. Due to visitor restrictions, his family could not accompany him, complicating communication. Palliative care specialists play a crucial role in facilitating discussions about prognosis and goals of care, particularly in the absence of family presence. However, the limited availability of palliative care specialists underscores the need for all clinicians to receive serious illness communication training. Such training programs, incorporating COVID-specific communication scripts, help nephrologists improve their communication skills in discussing prognoses and delivering difficult news. The case also highlights the additional challenge of language barriers, with the patient requiring a language interpreter. Healthcare systems can implement telecommunication platforms and virtual interdisciplinary conferences to improve communication access for patients and families, particularly during crises like the COVID-19 pandemic. In this case, the lack of telecommunication capability and the unavailability of a Spanish interpreter hindered family communication during his conscious moments.

#### Case #2 (challenges in prognostication)

A 39-year-old woman with type 2 diabetes mellitus and kidney failure was hospitalized for respiratory distress due to COVID-19. Despite initially stable conditions, her health condition deteriorated, requiring ICU admission for acute pulmonary embolism and mechanical ventilation. The patient, unable to communicate, requested her brother to be updated on her condition. Prognosticating patient outcomes presents challenges due to the novelty of COVID-19 and the absence of validated predictive models for kidney failure patients. Physicians often struggle with discussing prognosis, especially in uncertain situations, leading to a ‘collusion of silence’ where prognostic conversations are avoided or unclear. Patients with kidney disease desire and benefit from transparent discussions about prognosis, yet such conversations remain infrequent. The lack of disease-specific predictive models for COVID-19 complicates prognostication further, leaving nephrologists without their usual tools. Communication frameworks suggest acknowledging uncertainty, validating emotions, providing information gradually, and confirming understanding through ‘teach-backs.’

#### Case #3 (advanced care directives)

An 82-year-old man with mild cognitive impairment and CKD faced challenges in dialysis decision-making amid the COVID-19 pandemic. Patient’s reluctance to formalize advanced care planning due to the perceived complexity of existing directives reflects a common issue encountered in patients with chronic conditions. Physician Orders for Life-Sustaining Treatment (POLST) or Medical Orders for Life-Sustaining Treatment (MOLST) forms may offer a more accessible alternative to advanced directives, facilitating timely, goal-concordant care, especially in nephrology crisis settings [65].

This case highlights the importance of exploring options and developing realistic care plans that align with patients’ preferences, particularly as care needs escalate. The ethical principle of respect for autonomy is crucial in ensuring patients like this can make decisions consistent with their values, unencumbered by external perspectives. Creative models, such as increased availability of palliative care consultation and virtual shared decision-making, are essential in crisis settings where in-person discussions are limited. Telemedicine allows for timely shared decision-making, even for patients lacking decision-making capacity due to cognitive impairment or critical illness.

Notably, the COVID-19 pandemic has led to increased use of telecommunication technologies, such as telehealth, to maintain care for dialysis patients while reducing in-person interactions. Though telecommunication has been crucial in ensuring continuity of care, it also presents challenges, including technical barriers, difficulties in assessing non-verbal cues, and maintaining personal connection. Nephrologists must adapt their communication strategies to ensure effective patient understanding and support during virtual consultations, making telecommunication a valuable yet evolving tool in patient care.

**Fig. 5:**
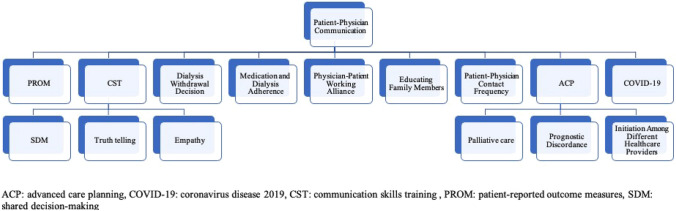
Summary of reported factors effective in patient-physician communication among patients with advanced kidney disease

Table 1 shows current barriers and potential solutions in patient-physician communication in advanced kidney disease. Additionally, a summary of all reported effective factors in patient-physician communication is provided in Fig. 5.

**Table 1:** Current barriers and potential solutions regarding patient-physician communication

Barriers	Solutions
*Patient-reported outcome measures (PROM)*	
- Patient misunderstanding of the purpose - Difficulty quantifying symptoms - Limited relevance of PROMs to individual needs - Passive approach to communication	- Improve patient education on PROMs’ purpose - Use adaptive or tailored PROMs - Train clinicians to interpret and act on PROM data - Incorporate real-time feedback mechanisms - Promote symptom descriptors or visual aids for better quantification
*Advanced care planning (ACP)*	
- Patients not ready to discuss end-of-life care - Focus on survival rather than death - Uncertainty about which doctor will provide care when seriously ill - Lack of communication on key end-of-life topics (e.g., prognosis, dying process, spiritual beliefs) Nephrology nurses not initiating ACP discussions	- Gradual introduction of end-of-life topics - Patient-centered communication training - Clarifying care continuity - Tailored end-of-life discussions - Enhance ACP training and role clarification for nurses
*Dialysis withdrawal initiation*	
- Lack of guidelines, prognostic tools, and training for dialysis withdrawal decisions - Absence of patient-reported outcome measures and quality of life assessments - Expectations from other specialties and difficulty initiating conversations - Fear of peer judgment in decision-making - Societal expectations and misperceptions about dialysis and kidney failure	- Develop guidelines and prognostic tools - Integrate patient-reported outcomes and quality of life measures - Communication and palliative care training among nephrologists - Promote a non-judgmental environment among peers - Public education
*Physician–patient working alliance*	
- Lack of a strong physician–patient working alliance (PPWA)	- Strengthen PPWA through regular communication and goal alignment - Integrate mental health (screen for depression) and social support assessments
*Family education*	
- Lack of family involvement in patient education	- Implement family-centered education programs - Foster collaborative decision-making - Provide ongoing family support
*Patient-physician contact*	
- Limited patient-physician contact - Lack of evidence on the ideal frequency	- Personalized follow-up plans - Utilize telemedicine and remote monitoring - Enhance self-management and patient empowerment - Multidisciplinary Team Involvement
*COVID-19*	
- Visitor restrictions - Limited availability of palliative care specialists - Uncertainty in prognostication - Telecommunication challenges	- Refine and enhance telecommunication capabilities - Train clinicians in serious illness communication - Promote interdisciplinary collaboration - Acknowledge prognostic uncertainty

## Conclusion

Training programs like NephroTalk highlight the significance of patient-physician communication in nephrology, especially regarding kidney failure diagnosis [21]. These initiatives tried to empower nephrologists with the skills necessary for navigating difficult conversations, including kidney failure, dialysis initiation or withdrawal, and end-of-life care discussions. While these agendas have been shown to improve physicians’ confidence and preparedness, deeper investigations suggest that the translation of training to long-term improvement in patient outcomes requires further research. Studies indicate moderate short-term effects, but the sustainability and direct impact on patient satisfaction and quality of life remain to be thoroughly evaluated [26].

This review emphasizes the importance of engaging patients through shared decision-making and advanced care planning and acknowledging patient autonomy [36]. However, barriers such as physicians’ reluctance to initiate uncomfortable conversations and misconceptions about patient readiness present significant challenges [49]. To improve shared decision-making and advanced care planning in nephrology care, efforts must address these barriers by creating an environment where open, empathetic dialogues are encouraged, ensuring that patient values and preferences guide clinical decisions [14].

Integrating patient-reported outcome measures in evaluating care quality and facilitating patient-centered communication in dialysis settings is a debated concept. While patient-reported outcome measures hold the potential for standardizing patient-reported information, there is a gap between the intended purpose and actual application. Misunderstandings among patients regarding the role of patient-reported outcome measures, coupled with a lack of awareness among clinicians on how to use these tools effectively, undermine their effectiveness [19]. Critical to enhancing the utility of patient-reported outcome measures is the need for comprehensive education for patients and healthcare providers, alongside system-level adjustments to seamlessly incorporate patient-reported outcome measures into routine clinical practice.

Despite advancements in communication skills training and implementing tools like patient-reported outcome measures, significant challenges persist in ensuring effective patient-physician communication in nephrology. This review identified a need for more targeted research to bridge these gaps, including studies on the long-term impact of communication training programs and strategies to increase the meaningful use of patient-reported outcome measures in clinical settings. Addressing systemic barriers to advanced care planning and shared decision-making, such as time constraints and cultural and institutional resistance, is crucial for improving communication and patient care outcomes in advanced kidney disease.

This review features the multidimensional nature of patient-physician communication in nephrology, emphasizing the critical need for improved communication skills training, effective use of patient-reported outcome measures, and overcoming barriers to advanced care planning and shared decision-making. While progress has been made, moving forward necessitates a collaborative effort from healthcare providers, researchers, and policymakers to fully realize the benefits of patient-centered communication in improving the care and quality of life for patients with advanced kidney disease.

## Data Availability

Data sharing not applicable to this article as no datasets were generated or analyzed during the development of this review article.
